# In vitro antimicrobial potential of extracts and phytoconstituents from *Gymnema sylvestre* R.Br. leaves and their biosafety evaluation

**DOI:** 10.1186/s13568-017-0416-z

**Published:** 2017-06-05

**Authors:** Daljit Singh Arora, Henna Sood

**Affiliations:** Microbial Technology Laboratory, Department of Microbiology, Guru Nanak Dev University, Amritsar, 143005 India

**Keywords:** Antimicrobial, Biosafety, *Gymnema sylvestre*, Medicinal plants, Minimum Inhibitory Concentration, Phytoconstituents

## Abstract

The in vitro antimicrobial screening of *Gymnema sylvestre* leaves against 13 test pathogens established its broad spectrum activity with average inhibition zone ranging from 14 to 23 mm. The antimicrobial activity of the classically- optimized aqueous extract was enhanced up to 1.45 folds, when subjected to statistical optimization using Response Surface Methodology (RSM) and was thermostable. Ethyl acetate was found to be the best organic extractant with *Klebsiella pneumoniae* 1 (31.5 mm) and *Staphylococcus epidermidis* (25.5 mm) being the most sensitive among Gram negative and Gram positive bacteria, respectively. Among the major group of phytoconstituents detected, tannins were the most abundant followed by flavonoids and phytosterols, while triterpenes were absent. Flavonoids and cardiac glycosides exhibited a broad range of antimicrobial potential, with inhibition zone ranging from 13 to 35 mm, where *Candida albicans* was the most sensitive organism. Ethyl acetate extract showed better potency with lowest Minimum inhibitory concentration (0.1–1 mg ml^−1^) than the aqueous extract (1–3 mg ml^−1^) and all partially purified phytoconstituents (0.1–10 mg ml^−1^). The ethyl acetate extract and flavonoids were highly potent, as they exhibited a total activity potency ranging from 41.4 to 1045 ml g^−1^. Time kill studies revealed their microbicidal action, where ethyl acetate extract had a kill time from 0 to 12 h. However, among phytoconstituents, flavonoids were the most effective (0–8 h). The MIC and time kill study was also compared to that of standard antibiotics. These findings indicate that *Gymnema sylvestre* can be a potential source for development of leading metabolites against pathogens of clinical importance like *Pseudomonas aeruginosa, Candida albicans, Escherichia coli, Staphylococcus aureus* etc. They were neither mutagenic nor cytotoxic, as revealed by Ames and MTT assay.

**Electronic supplementary material:**

The online version of this article (doi:10.1186/s13568-017-0416-z) contains supplementary material, which is available to authorized users.

## Introduction

Antimicrobials play a significant role in the world of medical health and have revolutionized it in different ways. For the past 50 years, antimicrobial drugs have saved countless lives from life-threatening diseases and have eased patients’ suffering. In the present era, the treatment of infections is once again becoming increasingly convoluted because microorganisms are gaining resistance to these “wonder drugs” worldwide (Cantas et al. 2013; Davies and Davies 2010). Therefore, there is an urgent need to search novel bioactive molecules, their chemical characterization and understand the mechanism of action so as to minimize the use of such antibiotics for prophylactic and therapeutic purposes (Savoia 2012). Moreover, for fungi and protozoa, the current chemotherapeutic options are very limited and far from ideal, especially owing to their undesirable side effects or toxicity. This medical crisis has drawn the attention of the scientific community towards studies on the potential of plant-derived substances, an untapped resource of antimicrobial chemotypes, which have long historical usage in traditional pharmacopoeia used in different countries. Plants are rich in several secondary metabolites and are major source of chemical diversity; therefore, they are a potential source of new drugs (Savoia 2012). Medicinal plants have played a critical role in the health and well-being of humans both prophylactically and therapeutically for controlling diseases (Upadhyay et al. 2014). Their potential has been well documented in the literature (Ferreira et al. 2006; Arora and Kaur 2007; Onsare et al. 2013; Teiten et al. 2013; Aumeeruddy-Elalfi et al. 2016).

*Gymnema sylvestre* R.Br. popularly known as Gurmar, is one such valuable medicinal plant belonging to the family Apocynaceae of the order Gentianales. It is mostly found in India and is also known as “miracle fruit”. This plant possesses many more bioactive properties such as antimicrobial, larvicidal, antiviral, hypolipidemic, anticancer, antioxidant activity. Thus, the plant has been studied to provide scientific validation to its traditional usage and optimize its antimicrobial activity, both classically and statistically, using different extraction strategies and other physicochemical parameters. In addition, the aqueous and ethyl acetate extract has also been studied for its Minimum Inhibitory Concentration (MIC), Viable Cell Count (VCC) studies. The plant material was also assayed for the presence of various phytoconstituents and tested individually for their antimicrobial potential. In order to be considered as a potential drug candidate, the biosafety aspect of the plant extract and its phytoconstituents has also been evaluated through Ames test and MTT assay.

## Materials and methods

### Plant material and its extract preparation

The chemicals used in this work were purchased from Hi-Media, Mumbai, India. The plant *Gymnema sylvestre* leaves were procured from the local market of Amritsar, Punjab. It was deposited in the Department of Botanical and Environmental Sciences, Guru Nanak Dev University, Amritsar vide accession no. 766–768 Bot. & Env.Sc., Dated 02/07/2015. The plant material was surface sterilized as described previously by Sood et al. (2015). The aqueous extract was then prepared and the filtrate was tested for its antimicrobial activity by Agar well Diffusion Assay (ADA) (Sood et al. 2015).

### Test microorganisms

The reference bacterial strains, such as *Enterococcus faecalis* (MTCC 439), *Staphylococcus aureus* (MTCC 740) and *Staphylococcus epidermidis* (MTCC 435) as Gram positive bacteria; *Escherichia coli* (MTCC 119), *Klebsiella pneumoniae* 1 (MTCC 109), *Klebsiella pneumoniae* 2 (MTCC 530), *Pseudomonas aeruginosa* (MTCC 741), *Salmonella typhimurium* 1 (MTCC 98), *Salmonella typhimurium* 2 (MTCC 1251) and *Shigella flexneri* (MTCC 1457) as Gram negative bacteria and yeast strains such as *Candida albicans* (MTCC 227) and *Candida tropicalis* (MTCC 230) were obtained from Microbial Type Culture Collection (MTCC), Institute of Microbial Technology (IMTECH), Chandigarh, India. A clinical isolate, i.e., Methicillin-resistant *Staphylococcus aureus* (MRSA) was procured from Post Graduate Institute of Medical Education and Research (PGIMER), Chandigarh, India.

### Antimicrobial screening of the aqueous plant extract

Ten percent aqueous extract of the plant material was tested for its antimicrobial activity. A clinical isolate (MRSA), 3 Gram positive and 7 Gram negative bacteria were activated by growing in the Nutrient broth for 4 h, whereas, 2 yeast strains, i.e., *Candida albicans* and *Candida tropicalis* were grown in Yeast Malt extract Broth (YMB) and Saboraud broth respectively. The turbidity of these actively growing suspensions was standardized to 0.5 McFarland standards (Sood et al. 2015). The screening was performed using Agar diffusion assay (ADA), where autoclaved distilled water was taken as a negative control. The experiment was performed in duplicates and repeated several times.

### Optimization of physiochemical parameters using one-factor-at-a-time (classical) approach

Various physiochemical parameters such as concentration (5–30%), extraction temperature (30–100 °C), extraction time (20–240 min), pH (natural, 3, 7 and 9) and filtration strategies (Whatman filter paper No. 1, muslin cloth, normal filter paper and centrifugation at 10,000*g* for 15 min) were optimized for obtaining the best antimicrobial activity.

### Statistical optimization of the physiochemical parameters by Response Surface Methodology (RSM) using Box–Behnken design

On the basis of earlier optimization studies carried out using one- factor-at- a- time (classical) method, parameters such as extraction temperature, time and extract concentration were taken as independent variables for the optimization by RSM using Box–Behnken design, as described earlier in Sood et al. (2015). Each variable was studied at three levels (−1, 0, +1); for temperature these were 40, 50 and 60 °C; extraction time: 20, 40 and 60 min, and concentration: 10, 15 and 20%.

### Thermostability studies of the aqueous extract

Fifteen percent aqueous plant extract, initially prepared at 40 °C, was tested for its thermostability by exposing it to a temperature range of 60–100 °C for 1 h. One set was kept as an untreated control. Antimicrobial activity of these sets was evaluated in terms of zone of inhibition and thereby percent loss, with respect to untreated control, was calculated.

### Determination of best organic extractant

Five organic solvents, i.e., chloroform, hexane, ethyl acetate, butanol and methanol were used for the extract preparation. 15% aqueous extract was shaken vigorously in a separating funnel three times with equal volume of the solvent. The pooled organic layer was concentrated in a rotary evaporator at 45 °C and the residue was reconstituted in 30% DMSO. A quantity of 100 µl of each organic extract was screened for its antimicrobial activity against the thirteen test organisms by agar well diffusion assay, where 30% DMSO was taken as a negative control.

## Phytochemical analysis of *Gymnema sylvestre*

### Qualitative analysis

Qualitative analysis for the detection of phytoconstituents in the powdered plant material was carried out by standard methods, with partial modifications, according to Arora and Onsare (2014c); Kaur and Arora (2009) as follows:

*Alkaloids* They were detected using Wagner’s, Meyer’s and Hager’s reagents separately. The tests were scored positive on the basis of brown precipitates, yellow precipitates and turbidity respectively. *Flavonoids* Occurrence of pink or magenta red coloration, magenta coloration, bulky white precipitate and dark green precipitate, respectively in Shinoda test, Zinc–hydrochloride reduction, lead acetate and ferric chloride tests was considered as a positive result. *Saponins* Formation of froth upon vigorous shaking indicated a positive test. *Tannins* Appearance of brownish–green/blue–black coloration and gelatinous/bulky white precipitation in ferric chloride test and lead acetate test respectively, is a positive indication. *Cardiac glycosides* These were detected using Keller Killiani test, where formation of reddish brown ring at the interface indicated their presence. *Terpenoids* Presence of triterpenes was detected using Salkowski’s test, with Golden yellow coloration as positive indication. Diterpenes were detected using copper acetate test, where formation of emerald green color indicated positive. *Anthranol glycosides* 0.2 g of plant powder was suspended in 8 ml of 1 M HCl and hydrolyzed for 2 h. Treatment of 2 ml of the hydrolysate with 5% ferric chloride solution, then an equal volume of benzene, which was then separated and treated with 10% ammonium solution, determined a formation of rose pink in ammonical layer in a positive case. *Phytosterols* Were detected using Libermann Burchard’s test and Salkowski test. *Coumarins* Their presence was assayed by adding 3 ml of 10% NaOH to 2 ml of the aqueous extract, where formation of yellow color indicated positive test.

### Quantitative isolation of phytoconstituents

The phytoconstituents, which were qualitatively detected in the plant, were quantitatively isolated by standard methods as described earlier (Kaur and Arora 2009; Samria and Sarin 2014; Arora and Onsare 2014c). The isolated phytoconstituents were studied for their antimicrobial potential by agar diffusion assay and thereby compared to standard antibiotics like gentamicin, chloramphenicol and amphotericin B (for yeast). Here, 30% DMSO was taken as a negative control (Additional file 1).

### Minimum Inhibitory Concentration (MIC)

Minimum inhibitory concentration of the aqueous extract, ethyl acetate extract and the active partially purified phytoconstituents (PPPs) was determined by agar dilution method.

Stock solutions were prepared and the minimum inhibitory concentration was determined using a range of concentrations, i.e., from 0.05 to 2% (equivalent to 0.5–20 mg ml^−1^) for aqueous extract, from 0.001 to 0.5% (0.01–5 mg ml^−1^) (organic) and from 0.001 to 1% (0.01–10 mg ml^−1^) (phytoconstituents) against test organisms, as described earlier by Onsare and Arora (2015). The MICs were compared with standard antibiotics (gentamicin and chloramphenicol) and amphotericin B, taken as positive controls.

### Total activity potency

Total activity potency (TAP) was determined according to Arora and Onsare (2014a) and was calculated by dividing the amount of extract in mg from 1 g plant material by the MIC of the test compound. The higher the total activity, the higher is the potency, expressed as ml g^−1^.

### Viable Cell Count (VCC) studies

Microbicidal/static activity of the aqueous and ethyl acetate extract as well as partially purified phytoconstituents (PPPs) was ascertained by viable cell count method, i.e., time kill assay, on the basis of time taken by compounds to induce complete killing of organisms. A stock solution was prepared and the study was performed according to Sood et al. (2015). Also, the time kill assay for standard antibiotics (gentamicin and amphotericin B) was determined. The experiment was performed in duplicate.

## Biosafety evaluation of *Gymnema sylvestre* leaves

### Ames mutagenicity test

The extracts and PPPs were subjected to Ames test by plate incorporation method to evaluate its mutagenicity. The inoculum was cultivated overnight in nutrient broth at 37 °C and serially diluted up to 10^−3^ dilution. Mutagenicity testing was performed according to Arora and Onsare (2014b) and Kaur et al. (2015), by adding cultivated culture of *Salmonella typhimurium* (MTCC 1251, IMTECH, Chandigarh) and equal volume of the extract (at its MIC concentration) to 5 ml of top agar containing 0.5 mM histidine–biotin mixture (1:1 ratio). Here, Sodium azide (5 µl of 17.2 mg ml^−1^) was used as a positive control.

### MTT toxicity assay

In order to check the level of cellular toxicity of the test extracts, MTT [3-(4,5-dimethylthiazol-2-yl)-2,5-diphenyl tetrazolium bromide] assay was performed according to method described in Arora and Onsare (2014c) and Sood et al. (2015), where 200 µl of the extract was added and incubated further for 24 h. The absorbance was measured at 590 nm using an automated microplate reader (Biorad 680-XR, Japan). The wells with untreated cells served as control.

### Data analysis

The experiments were performed in duplicate and repeated thrice. The data were analyzed by One-Way ANOVA at 5% level of significance. The statistical analysis was done by IBM SPSS Statistics Data editor Version 20.

## Results

### Antimicrobial activity of aqueous extract of *Gymnema sylvestre* leaves

Ten percent aqueous extract of *Gymnema sylvestre* (Gurmar) leaves exhibited a broad spectrum activity by inhibiting 9 out of 13 strains tested, with an average inhibition zone ranging from 14 to 23 mm (Table 1). *Pseudomonas aeruginosa* was the most sensitive microorganism (23.3 mm) followed by *Candida albicans*, the yeast strain, which exhibited an inhibition zone of 22.6 mm. Among the Gram negative, *Klebsiella pneumoniae* 1 (22.1 mm) was the most sensitive after *Pseudomonas aeruginosa*, followed closely by *Salmonella typhimurium* 2 (21.2 mm) and *Escherichia coli* (19.6 mm). *Staphylococcus aureus* and MRSA were the most sensitive among the Gram positive bacteria, with average zone of inhibition (20 and 19 mm) respectively. The other two Gram positive organisms, i.e., *Enterococcus faecalis* and *Staphylococcus epidermidis* were insensitive to the plant extract.

**Table 1:** Antimicrobial activity of 10% Aqueous extract of *Gymnema sylvestre* against some potential pathogens

Organisms	Zone of inhibition (mm)^a, b^
*Enterococcus faecalis*	–^c^
*Staphylococcus aureus*	20 ± 0.47
*Staphylococcus epidermidis*	–
*Escherichia coli*	19.6 ± 1.26
*Klebsiella pneumoniae* 1	22.1 ± 1.02
*Klebsiella pneumoniae* 2	–
*Pseudomonas aeruginosa*	23.3 ± 0.68
*Shigella flexneri*	14 ± 0.35
*Salmonella typhimurium* 1	14.3 ± 0.20
*Salmonella typhimurium* 2	21.2 ± 0.68
*Candida albicans*	22.6 ± 0.82
*Candida tropicalis*	–
MRSA	19 ± 0.68

^a^The Values are expressed as Mean ± Standard Errors of Means (SEM) for N = 5

^b^No significant difference was observed within rows at 5% significance level as revealed by One-Way ANOVA

^c^(–) → No zone of inhibition

### Optimization of physiochemical parameters using one-factor-at-a-time (classical) approach

#### Effect of concentration

The antimicrobial activity increased with an increase in concentration of the extract up to 15% and thereafter, only a marginal increase up to 30% was noted. However, *Salmonella typhimurium* 1, insensitive at 5%, was sensitive to higher concentrations. Therefore, 15% concentration was chosen as the optimal for best antimicrobial potency with average zone of inhibition (13.19 mm). At this optimal concentration, *Pseudomonas aeruginosa* was found to be the most sensitive organism (24.5 mm). Organisms such as *Klebsiella pneumoniae* 2*, Candida tropicalis*, *Enterococcus faecalis* and *Staphylococcus epidermidis* were found to be resistant, even at 30% concentration of the aqueous extract.

#### Effect of extraction temperature and time

A significant antimicrobial activity was observed at 30 °C, which rose to a maximum at 40 °C. Beyond this temperature there was only a marginal decline in activity up to 100 °C. *Shigella flexneri* and *Salmonella typhimurium* 1 completely lost their sensitivity to the extract prepared at boiling temperature. Therefore, 40 °C was chosen as the optimum temperature for further experimentation. The extract at optimal concentration (15%) and temperature (40 °C) was exposed to a range of time period for extraction, where 20 min was found to be the best optimized time period, since activity decreased with higher extraction time, though negligibly. For all the optimization parameters, *Pseudomonas aeruginosa* remained the most sensitive organism.

#### Effect of pH

Considerably good antimicrobial activity was exhibited at pH 3, which significantly increased at natural pH (5). At alkaline pH, strains such as *Salmonella typhimurium* 1, *Escherichia coli, Shigella flexneri* and MRSA lost their sensitivity, which were otherwise sensitive at acidic and natural pH values. Therefore, the extract at natural pH (5) showed the best potency.

#### Effect of different filtration strategies

Of the different filtration strategies tested, filtration through muslin cloth showed significant activity (13.53 mm), which varied more or less in case of other tested methods (11.3–12.03 mm). Therefore, filtration through muslin cloth was taken as the method of choice for the rest of the study.

### Statistical optimization of the physiochemical parameters by Response surface methodology (RSM) using Box–Behnken design

#### Fitting the model

The data obtained from the quadratic model equation was found to be significant. It was verified by F value and the analysis of variance (ANOVA) by fitting the data of all independent observations in response to surface quadratic model. The results for model F-value implies that the model is significant, which indicate it to be suitable to represent adequately the real relationship among the parameters used. R^2^ value for all the responses ranged from 90.4 to 96.5%, which showed suitable fitting of the model. The final predictive equations for each response: *S. aureus* (G_1_), *E. coli* (G_2_), *K. pneumoniae* 1 (G_3_), MRSA (G_4_) obtained are as follows:

$$\begin{aligned} {\text{G}}_{( 1)} &= { 4}0. 7 7 5+ 0.0 4 5 {\text{X}}_{ 1} + 0.00 1 {\text{X}}_{ 2} - 0. 8 50{\text{X}}_{ 3} + 0.0 3 1 {\text{X}}_{ 1}^{ 2} - 0.000 6 {\text{X}}_{ 2}^{ 2} \\ & \quad+ 0.0 10{\text{X}}_{ 3}^{ 2} + 0.00 2 {\text{X}}_{ 1} {\text{X}}_{ 2} - 0.0 20{\text{X}}_{ 1} {\text{X}}_{ 3} - 0.00{\text{X}}_{ 2} {\text{X}}_{ 3} \\ \end{aligned}$$

$$\begin{aligned} {\text{G}}_{( 2)} &= { 14}. 100 + \, 0. 5 30{\text{X}}_{ 1} + 0. 1 5 5 {\text{X}}_{ 2} - 0.0 7 5 {\text{X}}_{ 3} - 0.00 1 {\text{X}}_{ 1}^{ 2} - 0.00 2 6 {\text{X}}_{ 2}^{ 2} \\ & \quad - 0.000 2 {\text{X}}_{ 3}^{ 2} - 0.00 7 5 {\text{X}}_{ 1} {\text{X}}_{ 2} \; - 0.00 50{\text{X}}_{ 1} {\text{X}}_{ 3} + 0.00 3 7 {\text{X}}_{ 2} {\text{X}}_{ 3} \\ \end{aligned}$$

$$\begin{aligned} {\text{G}}_{( 3)}& = { 22}. 900 - 0. 3 30{\text{X}}_{ 1} - 0. 2 4 2 {\text{X}}_{ 2} + 0. 6 2 5 {\text{X}}_{ 3} - 0.0 4 4 {\text{X}}_{ 1}^{ 2} - 0.000 3 {\text{X}}_{ 2}^{ 2} \\ & \quad - 0.0 1 1 {\text{X}}_{ 3}^{ 2} + 0.00 5 {\text{X}}_{ 1} {\text{X}}_{ 2} + 0.0 30{\text{X}}_{ 1} {\text{X}}_{ 3} + 0.00 2 5 {\text{X}}_{ 2} {\text{X}}_{ 3} \\ \end{aligned}$$

$$\begin{aligned} {\text{G}}_{( 4)}& = { 65}. 4 7 5- 2. 6 4 5 {\text{X}}_{ 1} - 0. 3 3 9 {\text{X}}_{ 2} - 0. 7 7 5 {\text{X}}_{ 3} + 0.0 2 9 {\text{X}}_{ 1}^{ 2} + 0.00 2 {\text{X}}_{ 2}^{ 2} - 0.000{\text{X}}_{ 3}^{ 2} \\ & \quad - 0.00 3 {\text{X}}_{ 1} {\text{X}}_{ 2} + 0.0 40{\text{X}}_{ 1} {\text{X}}_{ 3} + 0.00 5 {\text{X}}_{ 2} {\text{X}}_{ 3} \\ \end{aligned}$$

The optimized values for factors were validated by repeating the experiment in triplicates.

#### Effect of different variables on *S. aureus*

R^2^ value was 94.2%. The response surface graph showed maximum predicted value of zone of inhibition for *S. aureus* (19 mm) at 40 °C, with an extraction time of 40 min at 15% concentration.

#### Effect of different variables on *E. coli*

R^2^ value was 90.4%. The maximum zone of inhibition for *E. coli* (19 mm) was obtained at 40 °C, with an extraction time of 40 min at 15% concentration.

#### Effect of different variables on *K. pneumoniae* 1

R^2^ value was 94.5%. The response surface graph showed that the maximum predicted value of zone of inhibition for *K. pneumoniae* 1 is 33 mm, when 15% extract was prepared at 50 °C for 30 min.

#### Effect of different variables on Methicillin- resistant *S. aureus* (MRSA)

R^2^ value was 92.4%. Zone of inhibition (in mm) was highest (24 mm) at 60 °C, with an extraction time of 40 min at 20%.

#### Validation of results

From the overall assessment, extraction temperature of 40–60 °C, concentration: 15–20% and extraction time: 30–40 min can be considered as the optimized conditions for antimicrobial activity. The F value and R^2^ value showed that the model correlated well with measured data and was statistically significant. To confirm the adequacy of the model, the verification experiments using optimum conditions were carried out. This resulted in enhanced antimicrobial activity by 1.17, 1.23, 1.45 and 1.20 folds for *S. aureus, E. coli, K. pneumoniae* 1 and MRSA respectively.

### Thermostability studies

The aqueous plant extract, prepared at 40 °C, was subjected to higher temperatures (60–100 °C) for 1 h so as to check its thermostability. 1 h exposure of the extract to 60–70 °C resulted in a maximum loss of activity of 23.52% against *Salmonella typhimurium* 1, though no such loss was observed against *Salmonella typhimurium* 2. At the higher temperatures (80–90 °C), a marginal loss in activity was observed for all the tested strains, except for *Salmonella typhimurium* 1, *Klebsiella pneumoniae* 1, *Shigella flexneri* and *Escherichia coli*, against which the extract completely lost its potential. The extract suffered a minimum loss of 8.82% against various test organisms used. Exposure to boiling temperature resulted in a maximum 100% loss for *Salmonella typhimurium* 1, *Klebsiella pneumoniae* 1, *Shigella flexneri, Escherichia coli* and minimum of 14.7% for *Salmonella typhimurium* 2.

### Determination of best organic extractant

Among the various organic solvents tested for extraction, ethyl acetate (23.53 mm) was found the most effective against the test organisms, with average inhibition zone ranging from 13.5 to 31.5 mm. *Klebsiella pneumoniae* 1 (31.5 mm) was found the most sensitive organism. The average inhibition zones of butanolic (22.61 mm) and hexane (21.84 mm) extract, however, did not differ significantly from the ethyl acetate extract. The methanolic extract was moderately active, with an average inhibition zone of 18.57 mm. Here, chloroform extract was found to be the least effective against the test pathogens (13.19 mm).

### Qualitative and quantitative phytochemical analysis

**Fig. 1:**
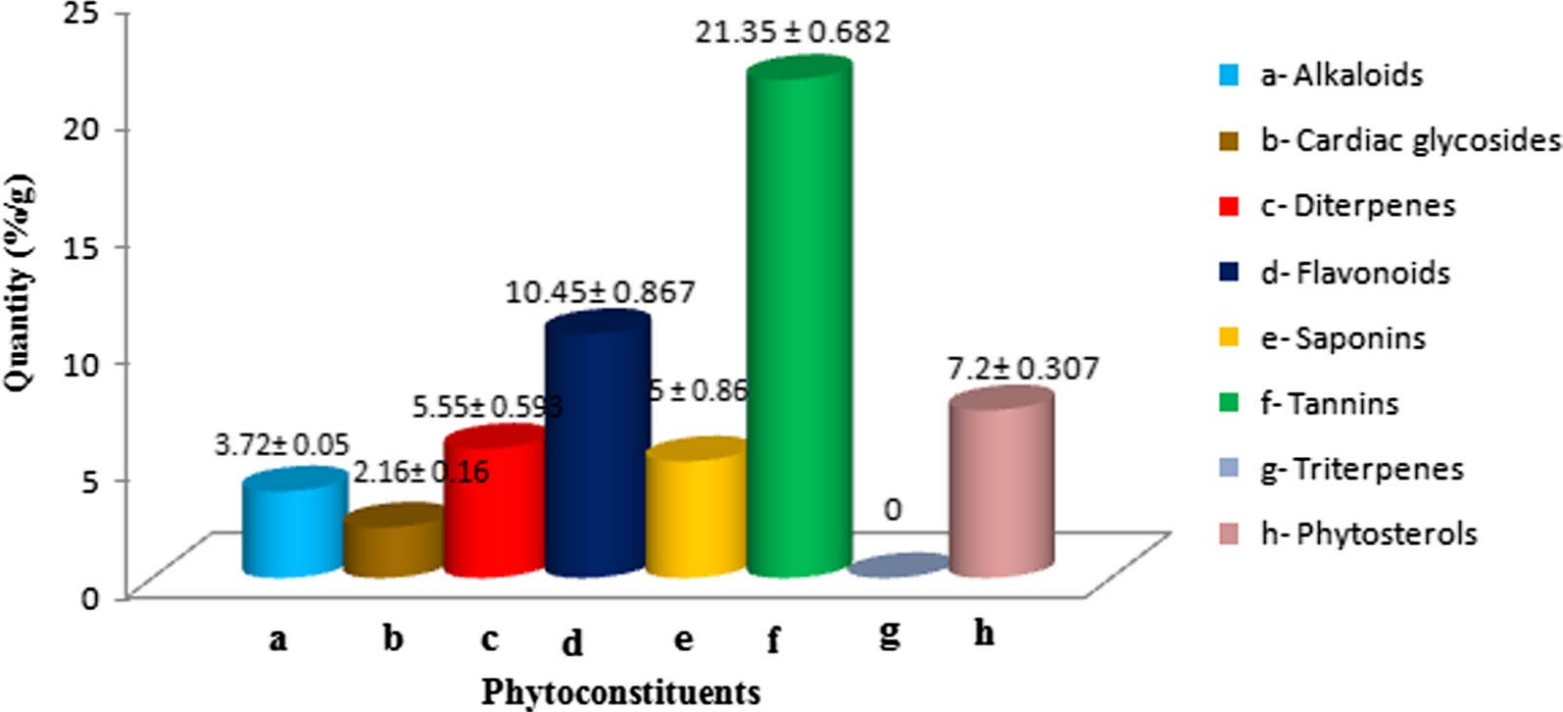
The concentration of various phytoconstituents of *Gymnema sylvestre* leaves (%/g). *The values are expressed as Mean ± SEM for N = 3. **No significant difference was observed within values at 5% significance level as revealed by One-Way ANOVA

Qualitative analysis by standardized methods was done for *Gymnema sylvestre*, so as to ascertain the various groups of phytochemicals present in the plant. Major groups such as alkaloids, flavonoids, saponins, tannins, cardiac glycosides, diterpenes, anthranol glycosides, phytosterols and coumarins were detected in the plant (Table 2), while, triterpenes could not be detected. Among the isolated phytoconstituents, their abundance was quite variable in respect of the plant material. Tannins were highly abundant (21.35%) of all the detected phytochemicals. Saponins and diterpenes were invariably present in equal abundance (5 and 5.55%), whereas, cardiac glycosides were present in lowest concentration (2.168%) (Fig. 1).

**Table 2:** Qualitative detection and antimicrobial activity of *Gymnema sylvestre* leaves

Phytoconstituents	Detected group	Stock solution (mg/ml)	Antimicrobial activity
Alkaloids		31	++ ^d^
Mayer’s reagent test	+^b^		
Hager’s reagent test	+		
Wagner reagent test	+		
Flavonoids		52.25	+++^c^
Shinoda test (Magnesium turnings)	-^a^		
Zinc–hydrochloride reduction test	+		
Lead acetate test	+		
Ferric chloride reagent test	+		
Saponins		50	++^d^
Froth test	+		
Tannins		142.3	-^f^
Ferric chloride reagent test	+		
Lead acetate test	+		
Cardiac glycosides		54.65	+++^c^
Keller-killiani test	+		
Terpenoids			
Triterpenes (Salkowski’s test)	–	NA	–
Diterpenes (Copper acetate test)	+	44.4	++^d^
Anthranol glycosides			
Borntrager’s test	+	ND	NA
Phytosterols		57.6	+^e^
Libermann Burchard’s test	+		
Salkowski’s test	+		
Coumarins	+	ND	NA

*NA* not applicable, *ND* not done

^a^Absent, ^b^ present, ^c^ most active, ^d^ active, ^e^ least active, ^f^ not active

Flavonoids and cardiac glycosides exhibited a significantly broad spectrum antimicrobial activity against the 13 standard bacterial and yeast strains. Flavonoids were active against 10 test organisms, with an average zone of inhibition ranging from 13.33 to 35 mm. Yeast such as *Candida albicans* was the most sensitive (35 mm) among all organisms. *Klebsiella pneumoniae* 2 (23 mm) and *Enterococcus faecalis* (19.66 mm) were most susceptible among Gram negative and Gram positive bacterial strains respectively. Strains like *Salmonella typhimurium* 1, *Staphylococcus aureus* and *Candida tropicalis* were, however, completely resistant to it. Cardiac glycosides also showed significantly high potential, with activity against 12 out of 13 test strains and average zone ranging from 13 to 20.33 mm. *Candida albicans* (20.33 mm) was the most sensitive, followed by *Staphylococcus epidermidis* (19.67 mm) and *Enterococcus faecalis* (19.33 mm). However, cardiac glycosides did not act against *Candida tropicalis.* Saponins followed up next in the queue as third active component with average inhibition zone ranging from 13.6 to 24.3 mm against 8 organisms. Alkaloids, phytosterols and diterpenes were found to be moderately active against the test pathogens, whereas, tannins were inactive against all the test strains (Table 3). The average inhibition zones of standard antibiotics, i.e., gentamicin, chloramphenicol and amphotericin B (for yeast) were also measured for reference. In case of *Candida albicans* and *Klebsiella pneumoniae* 2, flavonoids had inhibition zone comparable to the standard antibiotics.

**Table 3:** Antimicrobial activity of phytoconstituents isolated from *Gymnema sylvestre* leaves

Org^c^	Alkaloids	Flavonoids	Saponins	C. glycosides	Diterpenes	Phytosterols	Gentamicin	Chloramphenicol
Average zone of inhibition (mm)^b^								
SA	14.66 ± 0.33	0	0	15.33 ± 0.66	16 ± 0	14 ± 0	34.5 ± 0.50	26 ± 1.00
SE	14.33 ± 0.33	17.33 ± 0.33	15 ± 0	19.66 ± 0.33	0	13.33 ± 0.33	26.5 ± 0.50	28.5 ± 0.50
EC	0	15 ± 0.57	15.33 ± 0.33	15.66 ± 0.33	13.66 ± 0.33	0	31 ± 1.00	25 ± 0
EF	0	19.66 ± 0.66	0	19.33 ± 0.33	0	0	27.5 ± 0.50	26.5 ± 0.50
KP1	15.33 ± 0.33	19.66 ± 0.33	24.33 ± 0.33	16.66 ± 0.33	15.66 ± 0.33	13.66 ± 0.33	40.5 ± 0.50	38 ± 1.00
KP2	19.66 ± 0.33	23 ± 0.57	17.33 ± 0.33	16.66 ± 0.66	14 ± 0	15 ± 0	37.5 ± 1.50	26.5 ± 0.50
SF	0	13.33 ± 0.66	0	14.33 ± 0.33	13.33 ± 0.33	0	30.5 ± 0.50	27.5 ± 0.50
ST1	0	0	0	13 ± 0	12.66 ± 0.33	0	35 ± 0	23 ± 0
ST2	15 ± 0	20.33 ± 0.88	13.66 ± 0.33	17 ± 0	13 ± 0	0	43 ± 1.00	40.5 ± 0.50
PA	0	17 ± 0	20 ± 0.57	14.66 ± 0.33	13.33 ± 0.33	0	40.5 ± 0.50	28.5 ± 0.50
CA	19.66 ± 0.33	35 ± 0	19.66 ± 0.33	20.33 ± 0.88	0	17.33 ± 0.33	36.5 ± 0.50^a^	ND
CT	0	0	0	0	0	0	27.5 ± 0. 50^a^	ND
MRSA	13.66 ± 0.33	19 ± 0	18.33 ± 0.33	17.66 ± 0.33	15 ± 0	0	42 ± 0	39.5 ± 0.50

*ND* not done

^a^Amphotericin B

^b^Values are expressed as Mean ± SEM for N = 3

^c^ *Organisms* SA, *Staphylococcus aureus*; SE, *Staphylococcus epidermidis*; EC, *Escherichia coli*; KP1, *Klebsiella pneumoniae* 1; KP2, *Klebsiella pneumoniae* 2; SF, *Shigella flexneri*; ST1, *Salmonella typhimurium* 1; ST2, *Salmonella typhimurium* 2; PA, *Pseudomonas aeruginosa*; CA*, Candida albicans*; CT, *Candida tropicalis*; MRSA, Methicillin-resistant *Staphylococcus aureus*

### Minimum Inhibitory Concentration (MIC)

The MICs for aqueous extract, organic extract and the PPPs were worked out by agar dilution method. Their MIC values were organism-specific and quite variable with respect to each other. MIC values for aqueous extract ranged from 1 to 3 mg ml^−1^ where, *K. pneumoniae* 1 and *P. aeruginosa* exhibited the lowest value (1 mg ml^−1^). The organic, i.e., ethyl acetate extract exhibited significantly low MIC values as compared to PPPs and the aqueous extract. The PPPs had MIC values either comparable or slightly higher than the aqueous extract, where exception is diterpene, giving lower value (0.7 mg ml^−1^) for *S. aureus*. All PPPs showed a lower value (0.1–0.7 mg ml^−1^) than the aqueous extract (1 mg ml^−1^) for *K. pneumoniae* 1 and a similar effect was seen in cardiac glycosides against *Salmonella typhimurium* 2. Flavonoids and Saponins also showed lower MIC (1 mg ml^−1^) than the aqueous extract (3 mg ml^−1^) against *Candida albicans*. MRSA was interestingly more sensitive to flavonoids, cardiac glycosides and diterpenes, as it showed a lower MIC (0.1–0.7 mg ml^−1^) than the aqueous extract (3 mg ml^−1^). Flavonoids showed a significant potency with values ranging from 0.1 to 5 mg ml^−1^. Cardiac glycosides had values comparable to that of flavonoids, ranging from 0.7 to 5 mg ml^−1^. Saponins and diterpenes were effective only against 7–8 organisms, with values ranging from 0.5 to 5 mg ml^−1^. Resistant yeast strain such as *Candida tropicalis* was notably susceptible to the organic extract with a low MIC value of 1 mg ml^−1^ (Table 4).

**Table 4:** Minimum inhibitory concentration (MIC) of organic extract and phytoconstituents isolated from *Gymnema sylvestre* leaves

Org^d^	MIC (mg/ml)							
	Aq^a^	EA^b^	Flavonoids	Cardiac glycosides	Saponins	Diterpenes	Gentamicin	Chloramphenicol
SA	2.5	0.5	ND	3	ND	0.7	0.0002	0.01
SE	ND	0.7	3	5	5	ND	0.01	0.01
EC	2.5	0.5	3	3	3	ND	0.005	0.01
EF	ND	0.7	5	3	ND	ND	0.03	0.3
KP1	1	0.5	0.1	0.7	0.5	0.5	0.0002	0.01
KP2	ND	0.5	1	5	10	1	0.0005	0.001
SF	2.5	0.5	3	3	ND	3	0.005	0.01
ST1	3	0.5	ND	5	ND	ND	0.005	0.1
ST2	2.5	0.5	3	1	5	5	0.0003	0.001
PA	1	0.1	0.7	5	5	1	0.005	0.7
CA	3	0.1	1	3	1	ND	0.0003^c^	ND
CT	ND	1	ND	ND	ND	ND	0.1^c^	ND
MRSA	3	0.5	0.1	1	5	0.7	0.005	0.01

*ND* not determined

^a^Aqueous extract

^b^Ethyl acetate extract

^c^Amphotericin B

^d^ *Organisms* SA, *Staphylococcus aureus*; SE, *Staphylococcus epidermidis*; EC, *Escherichia coli*; KP1, *Klebsiella pneumoniae* 1; KP2, *Klebsiella pneumoniae* 2; SF, *Shigella flexneri*; ST1, *Salmonella typhimurium* 1; ST2, *Salmonella typhimurium* 2; PA, *Pseudomonas aeruginosa*; CA*, Candida albicans*; CT, *Candida tropicalis*; MRSA, Methicillin-resistant *Staphylococcus aureus*

### Total activity potency

Total activity potency (TAP) is the volume at which the components, extracted from 1 g of material, are diluted enough to maintain the same potency against the test pathogens. Potency of all the extracts was calculated against different organisms. The ethyl acetate (EA) extract showed a high potency ranging from 41.4 to 414 ml g^−1^, with more strength against Gram negative bacteria (which varied between 82.8 and 414 ml g^−1^) in comparison to 59.14–82.8 ml g^−1^ for Gram positive bacteria. Among the yeasts, *Candida albicans* (414 ml g^−1^) was more sensitive than *Candida tropicalis*. Interestingly, among all the extracts tested, only EA extract was potent against *Candida tropicalis* with a total activity of 41.4 ml g^−1^. Flavonoids showed an overall high strength, with total activity ranging from 20.9 to 1045 ml g^−1^. Gram negative bacteria (34.8–1045 ml g^−1^) were more sensitive than the Gram positive bacteria (20.9–34.8 ml g^−1^) and among the yeasts, only *Candida albicans* was sensitive (104.5 ml g^−1^). The total activity potency of cardiac glycosides ranged from 10.93 to 78.07 ml g^−1^, with *Klebsiella pneumoniae* 1 being the most sensitive showing a TAP value of 78.07 ml g^−1^. The saponins exhibited a total activity potency lower than 50 ml g^−1^, with exceptions like *Candida albicans* and *Klebsiella pneumoniae* 1, which were susceptible to a dilution of 50 and 100 ml g^−1^. Diterpenes showed a high potency against *Klebsiella pneumoniae* 1 (111 ml g^−1^) (Table 5).

**Table 5:** Total Activity Potency (TAP) of *Gymnema sylvestre* organic extract and its partially purified phytoconstituents

Organisms	Total activity potency (TAP) in ml/g				
	Organic^a^	Flavonoids^b^	C. glycosides^c^	Saponins^d^	Diterpenes^e^
SA	82.8	ND	18.21	ND	79.28
SE	59.14	34.83	10.93	10	ND
EC	82.8	34.83	18.21	16.66	ND
EF	59.14	20.90	18.21	ND	ND
KP1	82.8	1045	78.07	100	111
KP2	82.8	104.5	10.93	5	55.5
SF	82.8	34.83	18.21	ND	18.5
ST1	82.8	ND	10.93	ND	ND
ST2	82.8	34.83	54.65	10	11.1
PA	414	149.28	10.93	10	55.5
CA	414	104.5	18.21	50	ND
CT	41.4	ND	ND	ND	ND
MRSA	82.8	1045	54.65	10	79.28

*ND* not determined

^a^41.4 mg/g, ^b^104.5 mg/g, ^c^54.65 mg/g, ^d^50 mg/g, ^e^55.5 mg/g were used for the calculations

### Viable cell count (VCC) studies

**Fig. 2:**
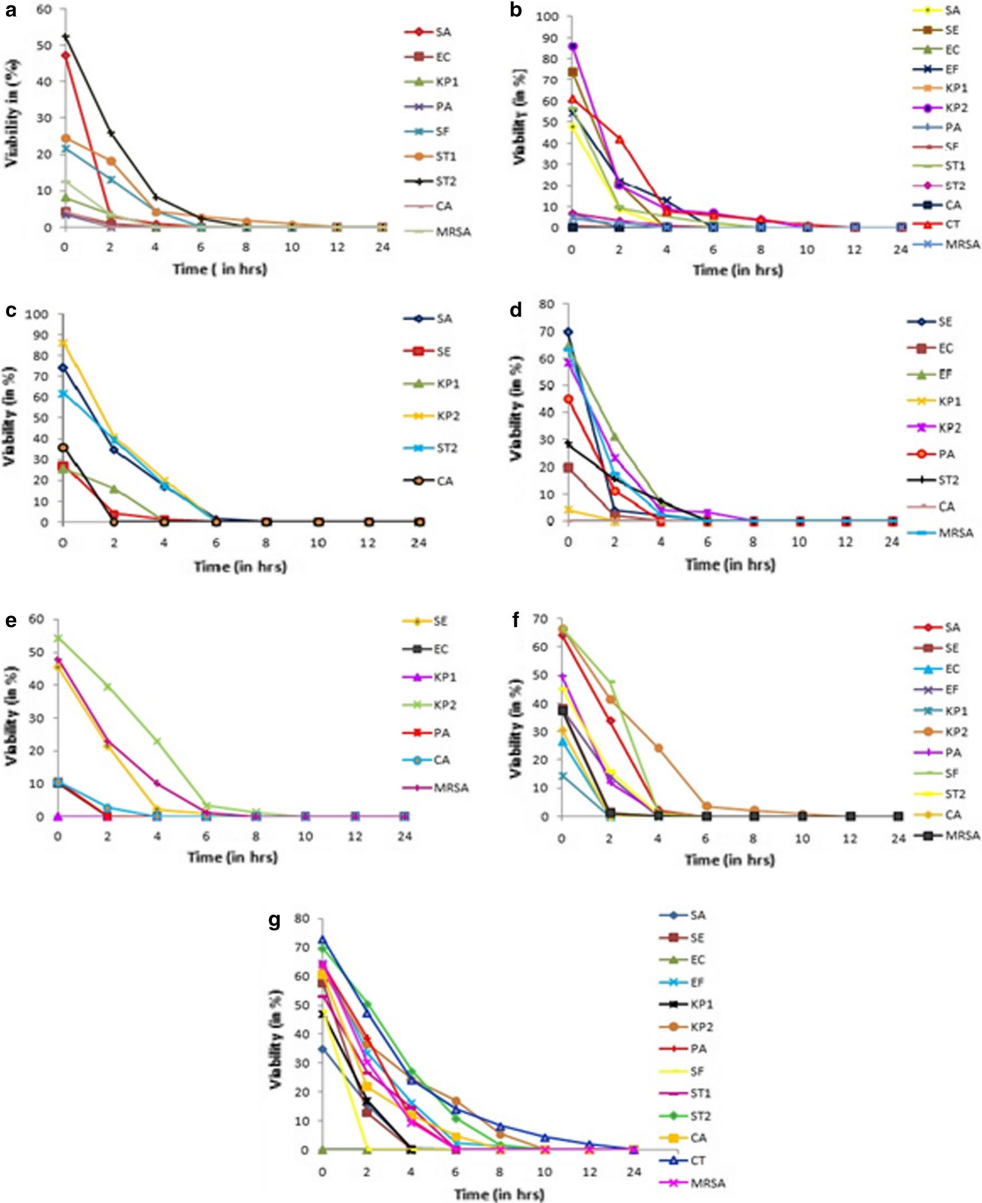
Viable cell count (VCC) studies for **a** Aqueous extract **b** Organic extract **c** Alkaloids **d** Flavonoids **e** Saponins **f** Cardiac glycosides of *Gymnema sylvestre* leaves and **g** Standard antibiotic Gentamicin (*Amphotericin B for yeast strains). SA, *Staphylococcus aureus*; SE, *Staphylococcus epidermidis*; EC, *Escherichia coli*; KP1, *Klebsiella pneumoniae* 1; KP2, *Klebsiella pneumoniae* 2; SF, *Shigella flexneri*; ST1, *Salmonella typhimurium* 1; ST2, *Salmonella typhimurium* 2; PA, *Pseudomonas aeruginosa*; CA*, Candida albicans*; CT, *Candida tropicalis*; MRSA, Methicillin-resistant *Staphylococcus aureus*

The test extracts had a variable effect on the organisms (Fig. 2a–f). The organic extract was more effective with a minor kill time than the aqueous extract, except in case of *Staphylococcus aureus*, where it took 8 h in comparison to 6 h for the aqueous extract. Organisms like *Pseudomonas aeruginosa* and MRSA took similar time period (2 and 4 h respectively) for both extracts. *Escherichia coli* and *Candida albicans* were highly sensitive to the organic extract and got killed instantaneously at 0 h, whereas, *Candida tropicalis* took 12 h to get completely killed. The partially purified phytoconstituents had a variable effect, when compared to the organic extract as well as the standard antibiotic gentamicin (Fig. 2g). *Escherichia coli* was killed instantaneously by the organic extract and gentamicin.

### Biosafety evaluation of *Gymnema sylvestre*

The aqueous extract, organic extract and the PPPs were subjected to Ames and MTT assay to evaluate their mutagenicity and cytoxicity. In aqueous extract, the number of revertant colonies in the positive control (652) was much higher than the test extract (19), whereas, in case of organic extract and phytoconstituents, no revertant colonies were observed when compared to the positive control (856). Since, the number of colonies in test was much less than the positive control this demonstrated non-mutagenicity of the extracts. MTT assay was also carried out. The absorbance for aqueous extract was 0.689 (test) and 0.752 (control), 0.728 (test) and 0.842 (control) for organic extract. For PPPs, the measured absorbance was 0.775 (alkaloids), 0.787 (flavonoids), 0.780 (cardiac glycosides) and 0.797 (saponins), which was comparable to 0.871 (control). Therefore, the extracts were found to be non-cytotoxic, with 91.62% (aqueous), 86.46% (organic), 88.97% (alkaloids), 90.35% (flavonoids), 89.55% (cardiac glycosides) and 91.5% (saponins) viable cells upon exposure to the extracts.

## Discussion

Antibiotics are one of the most important weapons in fighting bacterial infections and have provided huge benefit to the human health since their introduction. The successful use of any therapeutic agent is compromised by the microbial resistance, which is growing relentlessly, giving an uncertain outlook for the future use of antimicrobial drugs (Bhalodia and Shukla 2011; Davies and Davies 2010). For centuries, plants have been used throughout the world as drugs and remedies for various diseases. These drugs serve as prototype to develop more effective and less toxic medicines (Sharma et al. 2009). Therefore, a systematically deep study of the medicinal plants’ potential against pathogens could provide some solution to the burgeoning world health problem.

In an effort to expand the spectrum of antimicrobial agents from natural sources, the present study has been directed in such a way, to give the folklore use of medicinal plants, such as *Gymnema sylvestre*, a status of front-line treatment strategy. In a preliminary screening for its antimicrobial potential, it showed a broad spectrum potency, in line with previous studies on medicinal plants (Omoregie and Folashad 2013; Silva junior et al. 2009; Zampini et al. 2009). It is well known that *Pseudomonas aeruginosa* is a prototypical “multidrug resistant (MDR) pathogen” that is recognized for its ubiquity, intrinsically advanced antibiotic resistance mechanisms and association with serious illnesses. *Staphylococcus aureus* is associated with skin infections and food poisoning, whereas, *Candida albicans* is a causal agent of opportunistic, oral and genital infections in humans. Therefore, high sensitivity of these pathogens to the plant extract provided a great credence to the study. This encouraged the further optimization of various physiochemical parameters as well as the identification, isolation and study of responsible phytoconstituents.

In the classical optimization, the 15% was found to be the optimized concentration, which agrees well with the previous studies, where 4–20% concentration was optimal (Houshmand et al. 2013; Kaur and Arora 2009; Sood et al. 2015). The best/optimal concentration (15%) and extraction temperature of 40 °C agreed well with earlier studies, where 40–45 °C was the best (Akowuah and Zhari 2010; Chew et al. 2011). This may be due to increased solubility of the responsible metabolites at this temperature. Similarly, extraction time of 20 min is in line with earlier reports (Alberti et al. 2014; Mihaylova et al. 2015). Optimal pH (5.0) goes well with earlier observations reporting pH of 3.0–5.8 to be the best (Rahimi and Hasanloo 2016; Salama and Marraiki 2010) and may be attributed to better stability of the bioactive molecules under natural conditions. Filtration through muslin cloth had a slightly better result in this study and was taken up for further work. It may be due to larger pore size of the muslin cloth which allows the responsible active molecules to easily pass resulting in a better activity.

The statistical optimization by experimental design resulted in 1.17–1.45 fold increase in the antimicrobial efficacy in comparison to earlier experiments, where the antimicrobial potential was enhanced only by 1.23 fold (Fang et al. 2012) and 1.3 fold (El-Sersy et al. 2009). Apparently, statistical optimization using these parameters has not been previously reported for this plant. The aqueous extract was found to be relatively thermostable, as the extract retained its activity against all the test organisms at boiling temperature, except for four organisms. This property is quite useful during isolation, purification and processing of antimicrobial compounds from commercialization point of view. Ethyl acetate was found to be the best organic extractant with high inhibition zones in comparison to the aqueous extract. The fact that different solvent extracts vary in their potency may be explained by the solubility or insolubility of the active components into the solvent used (Linthoingambi and Singh 2013). It is well linked with the observation in this study as organisms like *Candida tropicalis*, *Enterococcus faecalis*, *Staphylococcus epidermidis* and *Klebsiella pneumoniae* 2 were sensitive to the organic extract, but not to the aqueous extract and/or phytoconstituents. The qualitative tests showed the presence of major class of phytoconstituents correlating well with the earlier studies on *Gymnema sylvestre* leaves, where more or less similar groups were reported (Murugan et al. 2012; Wani et al. 2012). This variation may be attributed to the use of different solvents in the reported literature. It throws light on the fact that the detection of phytoconstituents solely depends on the type of solvent used for isolation and the protocol followed. Quantification of the phytochemicals indicated tannins as the most abundant, which goes well in agreement with similar work on other plant species (Kaur and Arora 2009; Arora and Onsare 2014b). Tannins, however, did not possess any antimicrobial potential, also observed in case of *Moringa oleifera* seed coat and stem bark (Arora and Onsare 2014a, b). The relative abundance of other phytoconstituents is in consonance with previous studies on various solvent extracts of *Gymnema sylvestre* leaves (Naidu et al. 2013; Singh and Deo 2014). Cardiac glycosides and flavonoids showed the broad spectrum antimicrobial activity which is quite encouraging and agrees with studies on *Moringa oleifera* (Arora and Onsare 2014a, b). These findings are well supported by the fact that the phytochemicals are natural bioactive compounds that plants produce to protect themselves and recent researches have demonstrated their role in disease prevention in humans (Saxena et al. 2013). The MIC values of the aqueous extract, organic extract and the phytoconstituents supports the results of Agar well diffusion assay (ADA). These results are better than those obtained in earlier studies on medicinal plants, where it ranged from 5 to 15 mg ml^−1^ (Sood et al. 2015), 20–60 mg ml^−1^ (Kaur and Arora 2009), 8–14.2 mg ml^−1^ (Oskay et al. 2009). The low MIC values of organic extract may be attributed to good extractability and synergistic effect of active constituents in the particular solvent. Further, the total activity potency (TAP) values observed in this study were proportional to the MIC values of each extract against the test organism. The organisms showing lowest MIC showed the highest TAP and vice versa. Overall, the organic extract showed the highest strength against test organisms as compared to the phytoconstituents. The potential of flavonoids has been highlighted from this study, as they showed a higher potency than the organic extract and rest of the phytoconstituents against *Klebsiella pneumoniae* 1, MRSA (1045 ml g^−1^) and *Klebsiella pneumoniae* 2 (104.5 ml g^−1^).

In addition to the above studies, the extracts and phytoconstituents were also subjected to viable cell count studies, i.e., time kill assay. It is a more powerful prediction tool than MIC for determining the antimicrobial action of plant extracts, as it depicts time related rate of bactericidal activity (Ojo et al. 2013; Ruppen and Sendi 2015). Encouragingly, no re-growth was observed in any test organism against all the test extracts, i.e., the extracts exhibited bactericidal nature, which will be quite useful when considered for drug development purpose. Organic extract was found to be the most efficient of all with lesser kill time (0–12 h), whereas, flavonoids were most effective among PPPs, as they took from 0 to 8 h for complete killing. The efficiency of flavonoids was better or comparable to the kill time of standard antibiotic gentamicin (2–24 h) as well as other PPPs. It was observed that the kill time of test extracts for organisms like *Pseudomonas aeruginosa, Enterococcus faecalis, Klebsiella pneumoniae* 1*, Salmonella typhimurium* 2 and *Candida albicans* was better than the kill time of gentamicin. It was equal for one or more compounds in case of *Staphylococcus epidermidis, Klebsiella pneumoniae* 1*, Klebsiella pneumoniae* 2*, Pseudomonas aeruginosa* and MRSA, thus providing further credence to the study. The study also holds significance as the organic extract was more effective than amphotericin B against *Candida tropicalis*, where the former took only 12 h for complete killing as compared to 24 h respectively. This time kill study revealed better results than the previous study on *Moringa oleifera* organic extract, where *Pseudomonas aeruginosa*, *Candida albicans* and MRSA were killed at 12, 24 and 10 h respectively (Arora and Onsare 2014b). Further, before taking a natural compound to the level of commercialization and intending it for human use, it is necessary to make sure of its non cytotoxic profile. The extracts used in this study were found to be non-cytotoxic and non-mutagenic as demonstrated by MTT assay and Ames test respectively. Such biosafety of some other medicinal plants as well as some fungi has also been demonstrated previously (Arora and Chandra 2011; Kasolo et al. 2011; Ping et al. 2013; Kaur et al. 2014).

This study concluded with highlighting the true antimicrobial potential of an important medicinal plant, i.e., *Gymnema sylvestre* (Gurmar) due to presence of wide variety of phytoconstituents, thus providing a platform for its usage at commercial level. The aqueous extract, organic extract and the phytoconstituents showed a broad spectrum activity against all the test pathogens, especially those with high clinical importance such as *Pseudomonas aeruginosa, Escherichia coli, Candida albicans* and a multidrug resistant pathogen Methicillin-resistant *Staphylococcus aureus* (MRSA). These test compounds, even in their partially purified form, were highly potent with low and quite low MIC values and lesser killing time. Most importantly these were found to be non-cytotoxic as well as non-mutagenic. This study has provided a better understanding of how physicochemical conditions can affect metabolite production and their isolation. These findings provide a strong background for further purification and exploitation of these compounds for pharmaceutical purposes, so as to develop drug leads against rising resistant microbes.

## Additional file

**Additional file 1.** Quantitative isolation of phytoconstituents.

## Abbreviations

ADA: Agar Diffusion Assay
MIC: Minimum Inhibitory Concentration
MTT: [3-(4,5-dimethylthiazol-2-yl)-2,5-diphenyl tetrazolium bromide]
MTCC: Microbial Type Culture Collection
MRSA: Methicillin-resistant *Staphylococcus aureus*
PPPs: Partially Purified Phytoconstituents
RSM: Response Surface Methodology
TAP: Total Activity Potency
VCC: Viable Cell Count Study
