# Supplementary material for: In vitro antimicrobial potential of extracts and phytoconstituents from Gymnema sylvestre R.Br. leaves and their biosafety evaluation
Source: AMB Express. 2017 Jun 5;7:115. doi: 10.1186/s13568-017-0416-z (PMC5459786; doi:10.1186/s13568-017-0416-z)
Supplement: Supplementary file 1 — Additional file 1. Quantitative isolation of phytoconstituents. [file 13568_2017_416_MOESM1_ESM.docx]

**Quantitative isolation of phytoconstituents**

The phytoconstituents which were qualitatively detected in the plant were quantitatively isolated by standard methods as described below:

**Flavonoids:** Two gram of powdered plant material was extracted with 40ml of 80% aqueous methanol under shaking conditions for 24h at 30°C. The filtrate, thus obtained, was evaporated on a rotary evaporator and the residual dry weight was determined as flavonoids. **Saponins**: Ten gram plant powder was extracted in 50ml of 20% aqueous ethanol for 30min under shaking condition, and was thereafter kept in a water bath for 4h at 55°C. The filtrate obtained was reduced to 20 ml volume in a water bath at 90 °C, followed by three times extraction with 20ml of diethyl ether. The aqueous portion was retained, pooled and extracted twice with 60ml butanol. The butanolic layer was pooled and washed two times with 10ml of 5% NaCl. The butanolic portion was then concentrated and dried to obtain a constant dry weight of saponins. **Cardiac glycosides:** Plant powder (2g) was extracted three times with 40 ml of methanol under shaking conditions at 30°C for 24hrs. The combined filtrates were evaporated to dryness and then defatted with petroleum ether for 24hrs. Following decantation, the dried residue was redissolved in 50% aqueous methanol and was extracted three times with chloroform. The filtrates were pooled and concentrated to obtain the dry weight as cardiac glycosides. **Diterpenes:** Two gram of the plant powder was extracted three times in 40ml of 50% ethanol at 30 °C for 24h. The collected filtrates were pooled and evaporated to dryness. The dried residue was weighed as diterpenes. **Tannins:** For isolation of tannins, 2g of plant powder was extracted five times with a mixture containing 10ml of 8% sodium carbonate (Na_2_CO_3_) and 20 ml distilled water in the ratio 1:15 (w/v). A 10ml volume of HCl and 20ml formaldehyde was added to the combined filtrate and kept under reflux for 30 min. The mixture was filtered in a pre weighed Whatmann paper**,** which was oven-dried to obtain a constant weight as tannins. **Phytosterols:** The isolation was carried out according to Samria and Sarin, 2014 with slight modifications. Here, 2g of powdered plant material was defatted three times in petroleum ether for 24h on a water bath. The defatted material was air-dried and hydrolyzed with 50ml of 30% HCl (v/v) for 4h. The sample was repeatedly washed with distilled water till pH 7 was obtained. The sample was then dried and then extracted three times with 40ml of benzene for 24h. The filtrates were combined and dried *in vacuo* to obtain the dried mass as phytosterols
